# Mitigation of drought stress in maize and sorghum by humic acid: differential growth and physiological responses

**DOI:** 10.1186/s12870-024-05184-4

**Published:** 2024-06-07

**Authors:** Mohamed E. Abu-Ria, Eman M. Elghareeb, Wafaa M. Shukry, Samy A. Abo-Hamed, Farag Ibraheem

**Affiliations:** 1 Botany Department, Faculty of Science, Mansoura University, Mansoura, 35516 Egypt; 2 Biology and Chemistry Department, Al-Qunfodah University College, Umm Al-Qura University, Al-Qunfodah, 21912 Saudi Arabia

**Keywords:** Abiotic stress, Physiology, Photosynthesis, Oxidative markers, Osmolytes, Antioxidants

## Abstract

**Background:**

Drought is a major determinant for growth and productivity of all crops, including cereals, and the drought-induced detrimental effects are anticipated to jeopardize world food security under the ongoing global warming scenario. Biostimulants such as humic acid (HA) can improve drought tolerance in many cereals, including maize and sorghum. These two plant species are genetically related; however, maize is more susceptible to drought than sorghum. The physiological and biochemical mechanisms underlying such differential responses to water shortage in the absence and presence of HA, particularly under field conditions, are not fully understood.

**Results:**

Herein, the effects of priming maize and sorghum seeds in 100 mg L^−1^ HA on their vegetative growth and physiological responses under increased levels of drought (100%, 80%, and 60% field capacity) were simultaneously monitored in the field. In the absence of HA, drought caused 37.0 and 58.7% reductions in biomass accumulation in maize compared to 21.2 and 32.3% in sorghum under low and high drought levels, respectively. These responses were associated with differential retardation in overall growth, relative water content (RWC), photosynthetic pigments and CO_2_ assimilation in both plants. In contrast, drought increased root traits as well as H_2_O_2_, malondialdehyde, and electrolyte leakage in both species. HA treatment significantly improved the growth of both plant species under well-watered and drought conditions, with maize being more responsive than sorghum. HA induced a 29.2% increase in the photosynthetic assimilation rate in maize compared to 15.0% in sorghum under high drought level. The HA-promotive effects were also associated with higher total chlorophyll, stomatal conductance, RWC, sucrose, total soluble sugars, total carbohydrates, proline, and total soluble proteins. HA also reduced the drought-induced oxidative stress via induction of non-enzymic and enzymic antioxidants at significantly different extents in maize and sorghum.

**Conclusion:**

The current results identify significant quantitative differences in a set of critical physiological biomarkers underlying the differential responses of field-grown maize and sorghum plants against drought. They also reveal the potential of HA priming as a drought-alleviating biostimulant and as an effective approach for sustainable maize and sorghum production and possibly other crops in drought-affected lands.

**Supplementary Information:**

The online version contains supplementary material available at 10.1186/s12870-024-05184-4.

## Introduction

Drought stress is a major limiting abiotic stress that threatens the growth and productivity of many crops worldwide. The drought-induced loss in crop productivity approached 34% in 2021 [1]. Unfortunately, the ongoing global warming and climatic changes are expected to intensify the incidence and severity of drought stress and agricultural water scarcity, eventually exacerbating loss in crop productivity, and threatening world food security [2, 3]. Plants experience drought stress when soil water availability in the rhizosphere falls below the limits for biomass production and efficient growth [4, 5]. Drought stress exerts drastic negative impacts on plant growth that detrimentally reduces final yield via altering the key plant physiological and biochemical mechanisms [6]. It induces osmotic stress, which disrupts water and nutrient uptake, cell turgidity, cell division, and redox balance [7, 8]. Drought stress also reduces leaf area, photosynthetic pigments, and photosynthetic CO_2_ assimilation [9]. Further, drought induces stomatal closure which elicits the excessive production of reactive oxygen species (ROS) that damage cellular components and may eventually lead to cell death [10].

Plants activate a wide array of defense mechanisms to cope with water shortage. Examples include investing more resources in improving root growth indices such as root length, dry matter accumulation, and root/shoot ratio to optimize their water uptake and water use efficiency [5, 11]. Plants also activate the biosynthetic pathways of various osmoprotectants such as soluble carbohydrates, proline, and total soluble proteins to minimize the negative impact of water shortage on the water status of the plant [12, 13]. Further, plants possess a well-organized antioxidative defense system that involves enzymatic (ascorbate peroxidase (APX), catalase (CAT), peroxidase (POD), and superoxide dismutase (SOD)) as well as non-enzymatic antioxidants such as phenols, flavonoids, and many other secondary metabolites to detoxify free radicals and ROS and thus minimize the deteriorative impacts of drought-induced oxidative stress [9, 14].

Maize (*Zea mays* L.) and sorghum (*Sorghum bicolor* L.) are among the stable crops grown around the world [15–17]. In fact, maize is the world’s third most important cereal crop, providing up to 30% of food calories to half of the world’s population [18]. Gluten-free sorghum is the fifth most essential cereal crop and is currently used as a raw material, oils, and biofuel production [19]. Both maize and sorghum serve as food sources for humans and livestock as well as industrial raw materials. They are also rich in phytochemicals essential in preventing chronic diseases [20, 21]. Interestingly, although sorghum and maize are genetically related, sorghum plants are more drought-tolerant than maize [3]. For example, drought stress can induce up to 66% reduction in maize yield compared to 39% in sorghum [22]. Drought stress significantly impacts the photosynthetic apparatus of maize and sorghum, inducing ROS accumulation, lipid peroxidation, and membrane injury [23]. Therefore, extensive research has been targeting better understanding of the growth and physiological responses of maize and sorghum under limited water supply. Results from this research can pave the route for discovery of effective and innovative strategies for improving drought tolerance in these critical crops and consequently increase their contribution to the food security of the continuous growing world population, particularly under the anticipated global warming scenario.

Humic acid (HA) priming has been suggested as an effective strategy for reducing the detrimental effects of abiotic stresses in plants by upregulating stress tolerance-related physiological processes, genes, and hormonal signaling pathways [24]. It is a major constituent of humic substances released from the biochemical decomposition of both plant and animal remains [25]. Several studies have applied HA as a priming agent to alleviate many abiotic stress factors such as drought, salinity, and heavy metals in many plant species [26–28]. HA improves the germination and growth via upregulation of endogenous phytohormones, photosynthesis, leaf water content, nutrient homeostasis, and antioxidant system under different stresses [29–32]. HA stimulative effects on growth and drought tolerance have been reported in several plant species, such as wheat [33], soybean [34], faba bean [35], and barley [36].

Most of the reported differences in the drought tolerance and the responses to humic acid between maize and sorghum are derived from studies on either greenhouse- or pot-grown plants. In addition, most of these studies were performed either on sorghum or maize in separate investigations. To our knowledge, investigations on the simultaneous responses of field-grown maize and sorghum to water shortage in the presence of HA are limited. In addition, despite the reported positive impact of HA on plant growth and drought resistance in many crops, the underlying physiological mechanisms of such promotive role are not fully understood. Herein, seeds of both maize and sorghum were primed in HA (100 mg L^−1^), simultaneously grown in the field under different levels of water shortage, and their growth and physiological responses were monitored. The hypothesis here is that the field-grown plants will maintain their differential tolerance capabilities to drought and such capabilities will be improved by HA treatment via induction of significant quantitative differences in growth traits and critical drought tolerance-related physiological mechanisms in these two genetically related species. Therefore, performing a comparative analysis of the impact of water limitation and HA on the growth indices, biomass accumulation and allocation, plant water status, photosynthetic carbon assimilation, and the antioxidant defense systems under different levels of drought stress in maize and sorghum would be a useful approach to decipher possible common and species-specific drought tolerance and HA-ameliorative mechanisms in these two genetically related species.

## Materials and methods

### Experimental area, soil, and plant materials

The current study was conducted on field-grown maize and sorghum plants at the nursery of the Botany Department, Faculty of Science, Mansoura University, Mansoura, Egypt, during the summer of 2023. Mansoura is located at 31.040948° N latitude and 31.378470° E longitude, with an elevation of 4.92 m above the mean sea level and an average annual precipitation of 5.26 mm. The climatic conditions over the study period were: relative humidity (57−75%), photoperiod (14 h light:10 h dark), and zero precipitation. The daily temperature during the study period varied over a range of 20–25 °C for minimum temperature and 26–38 °C for maximum temperature. The experimental area has soil with clay loam texture, pH of 7.98, CaCO_3_ 3.06%, organic matter 1.46%, available NPK in ppm (70 N, 26.26 P, 388.94 K), electrical conductivity 915.84 ppm, soluble cations in meq/100 g soil (Mg^2+^ 1.00, Ca^2+^ 2.02, Na^+^ 1.94, K^+^ 0.12), and soluble anions in meq/100 g soil (Cl^−^ 0.15, HCO_3_^−^ 0.72, SO_4_^2−^ 4.21). The soil had 38% soil water content at field capacity, permanent wilting point 19% and bulk density 1.32 g cm^− 3^.

Seeds of both maize (*Zea mays* L. SC 131) and sorghum (*Sorghum bicolor* L. H 306) were used in the current study. The maize seeds were provided by the Agricultural Research Center (Maize Research Institute, Sakha, Kafr El-Sheikh, Egypt), whereas the sorghum seeds were obtained from Sorghum Research Institute, Giza, Egypt. HA was obtained from Sigma–Aldrich company (CAT No. 53,680, St. Louis, MO, USA). The HA concentration (100 mg L^− 1^) was chosen after a preliminary experiment in which various HA concentrations (25, 50, 100, and 150 mg L^− 1^) and distilled H_2_O (as a control) were evaluated. The results revealed that 100 mg L^− 1^ HA significantly improved germination and seedling growth compared to the other concentrations (Supplementary Table 1).

### Experiment setup and drought treatments

Conventional tillage practices were followed for soil preparation before cultivation, and superphosphate (12.5%) at 60 Kg P_2_O_5_/ha and nitrogen fertilizer (33.5% N) at 288 Kg N/ha were added during soil preparation. The experiments were designed as split-plot design with three replicates. The field was divided into six main plots that were used for different soil moisture treatments: Control (well-watered, 0.38 m^3^ m^− 3^ volumetric water content (VWC), no HA priming), HA (well-watered, 0.38 m^3^ m^− 3^ VWC, HA priming), D_1_ (0.30 m^3^ m^− 3^ VWC, no HA priming), D_2_ (0.23 m^3^ m^− 3^ VWC, no HA priming), HA + D_1_ (0.30 m^3^ m^− 3^ VWC, HA priming), and HA + D_2_ (0.23 m^3^ m^− 3^ VWC, HA priming). The treatments: well-watered, D_1_ and D_2_ represented 100%, 80%, and 60% of field capacity, respectively. Also, the treatment “no HA priming” represented seeds primed in distilled H_2_O whereas “HA priming” represented seeds primed in 100 mg L^− 1^ HA. Plots contained 24 rows (3 m long and 50 cm apart). Each plot was subdivided into two subplots of 12 rows each for maize and sorghum. Uniform maize and sorghum seeds were sterilized for 5 min in ethanol (75%) and washed thrice with sterile distilled H_2_O, then divided into two sets. The first set of seeds of each species was soaked in distilled H_2_O, while the second set was soaked in 100 mg L^− 1^ of HA (C_9_H_9_NO_6_, 227.17, dissolved in distilled H_2_O) for 12 h at 25 ± 2 °C in a dark growth chamber. Seeds of maize and sorghum were hand-sewn (two seeds/hill and 25 cm between hills within a row). Plants were thinned to one plant/hill at the 2-leaf stage. Plots were kept weed-free manually and maintained at the field capacity until plants had 3–4 fully developed green leaves. Drought stress was then applied by managing irrigation to maintain the soil moisture at the desired levels of water field capacities for 20 days using a soil moisture meter (SM150 Soil Moisture Sensor; Delta-T Devices Ltd., UK).

### Plant sampling and measurements

At 37 days after sowing, two sets (three triplicates each) of plants from both maize and sorghum were carefully uprooted and collected from each treatment. The first set of plants was used for assessing growth indices including the shoot length, root length, root/shoot ratio, and leaf area. These plants were then divided into roots, stems, and leaves. The shoot and root lengths were assessed using a tape meter from the root/shoot junction point to the tip of the longest extended leaf and root, respectively, according to [37]. The root-to-shoot ratio was estimated as root length/shoot length. The leaf area of the uppermost fully expanded green leaf was estimated according to Palaniswamy et al. [38] using the equation [leaf area = leaf length × leaf breadth × 0.75]. Fresh weights of different plant organs were recorded using a digital balance. Plant organs were then oven-dried at 60 °C to constant weights and the dry weights were recorded. Using an electric stainless grinder, the dried leaves were ground into fine powder that was used for the determination of carbohydrates, proline, and non-enzymic antioxidants. Leaves from the second set of plants were instantly frozen in liquid N, saved at -80 °C, and used for the analysis of total soluble proteins, oxidative stress markers (MDA, H_2_O_2_), and antioxidant enzymes.

### Relative water content (RWC)

RWC of fresh leaves was estimated as described previously [39]. Leaf discs were collected from the fully expanded fresh leaves, and their fresh weight (FWT) was immediately recorded. Leaf discs were immersed in distilled H_2_O overnight, blotted dry, and their turgid weight (TWT) was recorded. The disks were then oven-dried at 80 °C for 24 h and weighed (DWT). RWC was calculated from the equation RWC (%) = (FWT-DWT)/(TWT-DWT) ×100.

### Measuring photosynthetic pigment and gas exchange parameters

Photosynthetic pigments in a known wight of the uppermost fully expanded leaf were extracted in dimethyl sulfoxide (DMSO) as described previously [40] and estimated using a Shimadzu UV-160 A spectrophotometer at 470, 645, and 663 nm. The pigments were calculated following [41, 42] and then expressed as mg g^− 1^ FWT. Photosynthetic gas exchange components [net photosynthesis rate (*P*_N_), transpiration rate (*E*), internal CO_2_ concentration (C_*i*_), and stomatal conductance (g_s_)] were measured in the fully expanded uppermost leaf using a portable photosynthesis system LCi-SD (Analytical Development Company, Hertfordshire, UK) on a clear sunny day between 9 AM to 11 AM. Stomatal resistance (SR) was calculated as 1/g_s_.

### Assessing the leaf hydrogen peroxide (H_2_O_2_) and malondialdehyde (MDA)

H_2_O_2_ and MDA in leaves were extracted by homogenizing a known amount of frozen leaf tissues in chilled trichloroacetic acid (TCA, 0.1%) at 4 °C. The homogenate was centrifugated at 12,000 rpm at 4 °C for 10 min and the supernatant was collected and used to estimate both H_2_O_2_ and MDA. H_2_O_2_ content was determined according to Alexieva et al. [43]. A 0.5 mL aliquot of leaf extract was added to 0.5 mL of phosphate buffer (100 mM, pH 7.0) and 2 mL of KI (1 M), mixed well, and the mixture was incubated for 1 h in the dark. The absorbance was then recorded at 390 nm. The H_2_O_2_ content was determined using a H_2_O_2_ standard curve and expressed as µmol g^− 1^ FWT. MDA concentration in the leaf extracts was assessed using thiobarbituric acid (TBA) according to Heath and Packer [44]. A mixture of 1 mL supernatant and 4 mL TCA (20%) containing TBA (0.5%) was heated for 30 min at 90 ˚C in a water bath. The mixture was then cooled and centrifugated at 10,000 rpm at 4 °C for 10 min. The absorbance of the supernatant was measured spectrophotometrically at 532 and 600 nm. The absorbance at 600 nm was subtracted from that at 532 nm, and the MDA content was calculated using an extension coefficient of 155 × 10^− 3^ µM^− 1^ cm^− 1^ and then was expressed as µmol g^− 1^ FWT.

### Determination of electrolyte leakage (EL) and membrane stability index (MSI)

The EL of leaf tissues was assessed using an EC-meter (HANNA Instrument, HI 8033) as described by Shi et al. [45]. Leaf discs were taken from fully developed fresh leaves, submerged in 30 mL of distilled H_2_O in vials, and incubated at 25 ˚C in the dark for 24 h. The initial electrical conductivity (EC1) was determined. The stoppered vials containing the leaf discs were placed in a boiling water bath for 20 min, cooled to 25 ˚C, and the EC was recorded again (EC2). EL was then determined by the equation [EL (%) = EC1/EC2 × 100], whereas MSI was calculated according to Sairam et al. [46] using the equation [MSI (%) = [1- (EC1/EC2)] × 100.

### Estimation of carbohydrates

Sucrose and total soluble sugars (TSS) were extracted by maintaining 0.1 g leaf dry tissues in ethyl alcohol (80%, v/v) for 12 h at room temperature [47]. Sucrose concentration was estimated by hydrolysing 0.1 mL alcoholic extract by 0.1 mL KOH (5.4 eq/L) in a water bath at 100 °C for 10 min. The hydrolysate was then mixed with 3 mL anthrone reagent, and the mixture was heated at 97 °C for 10 min then cooled to 25 °C. Using a spectrophotometer (Shimadzu UV-160 A spectrophotometer), the absorbance of the developed colour was recorded at 620 nm [48]. TSS was determined by mixing 0.1 mL of the ethanolic extract and 3 mL anthrone reagent, followed by heating at 100 °C for 10 min. The mixture was then cooled to room temperature, and the absorbance of the developed colour was recorded at 625 nm using a spectrophotometer [47]. The concentrations of sucrose and TSS were expressed as mg g^–1^ DWT. Total carbohydrates in 0.1 g dry weight were extracted in HCl (2.5 N) and spectrophotometrically determined using the anthrone reagent according to the method adopted by Hedge and Hofreiter [49]. A mixture of 1 mL extract and 4 mL freshly prepared anthrone was boiled for 8 min in a water bath and cooled down, and the absorbance of the produced color was determined at 630 nm using a spectrophotometer. Total carbohydrates were determined using a standard curve made by glucose and expressed as mg g^− 1^ DWT. The content of polysaccharides was determined as the difference between total carbohydrates and TSS.

### Estimation of leaf total soluble proteins (TSP) and proline

TSP extraction was carried out via macerating a known weight of the frozen leaf tissues in Tris–HCl buffer (pH 8, 0.2 M) [50]. The protein extract was centrifuged at 12,000 rpm for 10 min at 4 °C. The supernatant was collected, and 0.020 mL aliquots were mixed with 0.980 mL Coomassie Brilliant Blue G250 reagent and the optical density of the developed color was measured at 595 nm. TSP concentration was determined using bovine serum albumin (BSA) standard curve and then expressed as mg g^–1^ FWT [51]. Free proline in the dried leaf tissues was extracted in distilled H_2_O, following previously published reports [28, 52] and quantified as described by Bates et al. [53]. Proline concentration was determined by mixing 2 mL aqueous extract, 2 mL acid ninhydrin reagent, and 2 mL of glacial acetic acid. The mixture was then incubated in a boiling water bath for 1 h, cooled to room temperature, and the optical density of the developed color was measured at 520 nm. Proline concentration was measured using a proline standard curve and expressed as mg g^–1^ DWT.

### Estimation of non-enzymatic antioxidant compounds

Total flavonoids and phenols were extracted in methyl alcohol as described previously [54]. Total flavonoids were determined according to Marinova et al. [55]. A 1 mL aliquot of alcoholic leaf extract was added to 4 mL of distilled H_2_O and 0.3 mL NaNO_2_ solution (5%). The mixture was then kept at 25 °C for 5 min, and 0.3 mL of AlCl_3_ (10%) was added. After incubation at 25 °C for 6 min, 2 mL of NaOH (1 eq/L) and 2.4 mL of distilled H_2_O were added to the reaction mixture, and the absorbance of the developed color was measured at 510 nm. Using a quercetin standard curve, the concentration of total flavonoids was determined and expressed as mg quercetin equivalent g^− 1^ DWT. Total phenols were estimated as described previously [56]. Aliquots of 0.050 mL of alcoholic leaf extract were mixed with 0.40 mL Folin–Ciocalteu reagent and the mixture was incubated for 3 min at room temperature. Subsequently, 0.80 mL sodium carbonate (10%) was added, and the reaction mixture was incubated for 2 h in the dark at room temperature. The optical density of the developed color was determined using a spectrophotometer at 765 nm. Total phenols were determined using a gallic acid standard curve and were expressed as mg gallic acid equivalent (GAE) g^− 1^ DWT.

### Estimation of the activity of antioxidant enzymes

The extraction of catalase (CAT), ascorbate peroxidase (APX), peroxidase (POD), and polyphenol oxidase (PPO) was carried out by macerating known amounts of the frozen leaf tissues in ice-cold phosphate buffer (pH 7, 0.02 M). The homogenate was then centrifuged for 20 min at 12,000 rpm at 4 °C [57]. CAT activity was assayed by incubating 0.5 mL of the crude enzyme extract with 1 mL of phosphate buffer (pH 7, 0.01 M), 0.40 mL of H_2_O, and 0.50 mL of H_2_O_2_ (0.2 M) at 25 °C for 1 min [58]. Aliquots of 2 mL acid reagent (5% dichromate/acetic acid mixture, 1:3 v/v) were used to stop the enzymatic reaction, and the mixture was heated for 10 min. The optical density was measured at 610 nm. CAT activity was expressed in mmol H_2_O_2_ min^− 1^ g^− 1^ FWT. The activity of APX was assayed by measuring the decline in the absorbance at 290 nm using an extinction coefficient of 2.8 mM cm^− 1^ [59]. The enzymic reaction was initiated by adding 0.050 mL of the enzyme extract to 0.5 mL phosphate buffer (pH 7, 0.02 M), 0.075 mL H_2_O_2_ (2 mM), and 100 µL ascorbate (0.5 mM). APX activity was expressed in ascorbate mmol min^− 1^ g^− 1^ FWT. POD and PPO activities were determined spectrophotometrically at 420 nm according to Devi [60]. POD activity was determined by mixing 3 mL phosphate buffer (pH 6, 0.1 M) containing pyrogallol (0.05 M), 0.5 mL H_2_O_2_ (1%), and 0.1 mL of the crude enzyme extract, followed by incubation for 1 min at room temperature. Subsequently, 1 mL H_2_SO_4_ (2.5 N) was added to stop the enzymic mixture. One POD unit was defined as unit min^− 1^ g^− 1^ FWT. PPO was assayed by incubating 1 mL of enzyme extract with 2 mL of phosphate buffer (pH 7, 0.02 M) and 1 mL of pyrogallol (0.1 M) for 1 min at 25 °C. After incubation, the enzymic reaction was stopped by adding 1 mL of H_2_SO_4_ (2.5 N). One unit of PPO was expressed as unit min^− 1^ g^− 1^ FWT.

### Statistical analysis

All the experimental analysis was conducted in triplicate and the results are depicted as mean ± standard deviation (*n* = 3). The statistical analysis of the obtained data was performed using CoStat Version 6.3 software to test the effects of drought stress and humic acid factors. Figures were performed using GraphPad Prism version 9.0.2 (GraphPad Software, Inc., LA Jolla, CA, USA). Comparison among means of the tested treatments was performed by Fisher’s test at *p* ≤ 0.05. The principal component analysis (PCA) was created using JMP Pro software, while the Pearson correlation heatmap was performed with GraphPad Prism.

## Experimental results

### Effects of HA on plant growth traits and RWC of maize and sorghum plants under drought stress

Table 1 illustrates the interactive effect of drought stress and HA priming on the growth characteristics and RWC of maize and sorghum plants. Compared to the control plants, all growth traits were significantly (*p* ≤ 0.05) reduced with increasing drought stress in both species, albite at significantly different magnitudes. For instance, the D_2_ induced reductions of 18.7, 41.6, 58.7, 38.6, and 10.0% in shoot length, plant fresh weight, plant dry matter, leaf area, and RWC, respectively in maize. The corresponding D_2_-induced reductions in sorghum were 14.4, 16.1, 32.3, 24.7, and 12.2%, respectively. D_1_ also caused reductions in the above growth indices but to a significantly lower extent. In general, the drought-induced reduction in growth indices of maize was significantly (*p* ≤ 0.05) higher than in sorghum. In contrast, drought markedly enhanced the tested root traits (root length, root/shoot ratio) of both maize and sorghum and such enhancement generally increased as drought level increased. The percentages of D_2_-elicited increase were 37.5% and 23.2% in root length compared to 69.2% and 44.0% in root/shoot ratio in maize and sorghum, respectively. D_1_ also increased these two important root parameters but to significantly lower percentages. Under the well-watered conditions, HA priming increased most of the tested shoot and root traits, with root length, root/shoot ratio, and plant dry weight being the most responsive among the tested attributes. The HA-induced increases in these growth indices were 13.1, 9.1, and 20.0% in maize compared to 5.7, 3.4, and 14.3% in sorghum, respectively. Under drought stress, HA priming significantly (*p* ≤ 0.05) improved most of plant growth traits and RWC compared to stressed and unprimed treatment. Under D_2_ conditions, the intensity of HA-positive effects was prominent, particularly in root length and plant dry matter with 21.1% and 25.7% in maize, respectively compared to 7.7% and 24.1% in sorghum plants. Under D_1_ conditions, the corresponding HA-induced increases in these two important parameters approached 20.5% and 23.1% in maize compared to 5.1% and 17.7% in sorghum.

**Table 1:** Influence of HA on growth parameters and RWC of drought-stressed maize and sorghum plants

Treatments	Shoot length (cm)	Root length (cm)	Root/Shoot length (cm)	Plant FWT (g)	Plant DWT (g)	Leaf area (cm^2^)	RWC (%)
**Maize**							
C	275.43 ± 3.72b	23.73 ± 1.30e	0.086 ± 0.004d	1390.00 ± 20.86b	179.00 ± 8.26b	956.53 ± 32.98a	93.57 ± 1.57a
D_1_	242.96 ± 2.63d	28.96 ± 0.65d	0.119 ± 0.003c	998.33 ± 20.34d	112.83 ± 3.33d	806.25 ± 45.83b	91.07 ± 1.03b
D_2_	223.90 ± 1.25f	32.63 ± 0.99c	0.146 ± 0.004b	811.33 ± 15.61f	74.00 ± 0.50f	587.13 ± 52.92d	84.25 ± 1.37d
HA	285.56 ± 1.91a	26.83 ± 0.76d	0.094 ± 0.002d	1454.67 ± 19.81a	216.67 ± 9.57a	992.47 ± 33.94a	95.56 ± 0.47a
HA + D_1_	256.33 ± 2.40c	34.90 ± 1.28b	0.136 ± 0.006b	1074.00 ± 29.63c	138.90 ± 0.85c	810.43 ± 40.16b	93.64 ± 0.76a
HA + D_2_	236.13 ± 3.67e	39.53 ± 1.90a	0.168 ± 0.010a	873.33 ± 13.66e	93.00 ± 5.22e	723.26 ± 6.82c	88.61 ± 1.60c
**Sorghum**							
C	168.83 ± 1.04B	31.43 ± 0.60 F	0.186 ± 0.005 C	527.17 ± 11.03B	71.67 ± 3.33B	681.30 ± 35.85AB	90.23 ± 1.67AB
D_1_	161.80 ± 1.67 C	35.00 ± 0.30D	0.216 ± 0.002B	481.50 ± 1.73 C	56.50 ± 2.50 C	604.89 ± 26.91CD	84.44 ± 1.16D
D_2_	144.47 ± 0.50E	38.73 ± 0.31B	0.268 ± 0.003 A	442.50 ± 6.73D	48.50 ± 3.04D	513.05 ± 10.81E	79.14 ± 1.98E
HA	172.60 ± 1.35 A	33.32 ± 0.68E	0.193 ± 0.003 C	554.17 ± 6.53 A	81.83 ± 3.62 A	719.36 ± 21.44 A	91.51 ± 1.44 A
HA + D_1_	167.57 ± 0.51B	36.80 ± 0.72 C	0.220 ± 0.005B	520.83 ± 12.33B	66.50 ± 3.61B	643.78 ± 34.41BC	88.86 ± 0.76BC
HA + D_2_	159.33 ± 2.08D	41.73 ± 0.85 A	0.262 ± 0.005 A	480.00 ± 12.56 C	60.17 ± 3.33 C	566.25 ± 17.92D	86.84 ± 0.73CD

Means ± SD (*n* = 3) followed by different letters indicate significant responses whereas those followed by the same letters depict non-significant responses for the respective parameters at Fisher’s test (*P* ≤ 0.05) (uppercase letters for sorghum and lowercase letters for maize). C; Control, D_1_; drought level 1, D_2_; drought level 2, HA; humic acid, FWT; fresh weight, DWT; dry weight, RWC; relative water content

### Effects of HA on biomass allocation to leaves, stem, and root of maize and sorghum plants under drought stress

**Fig. 1:**
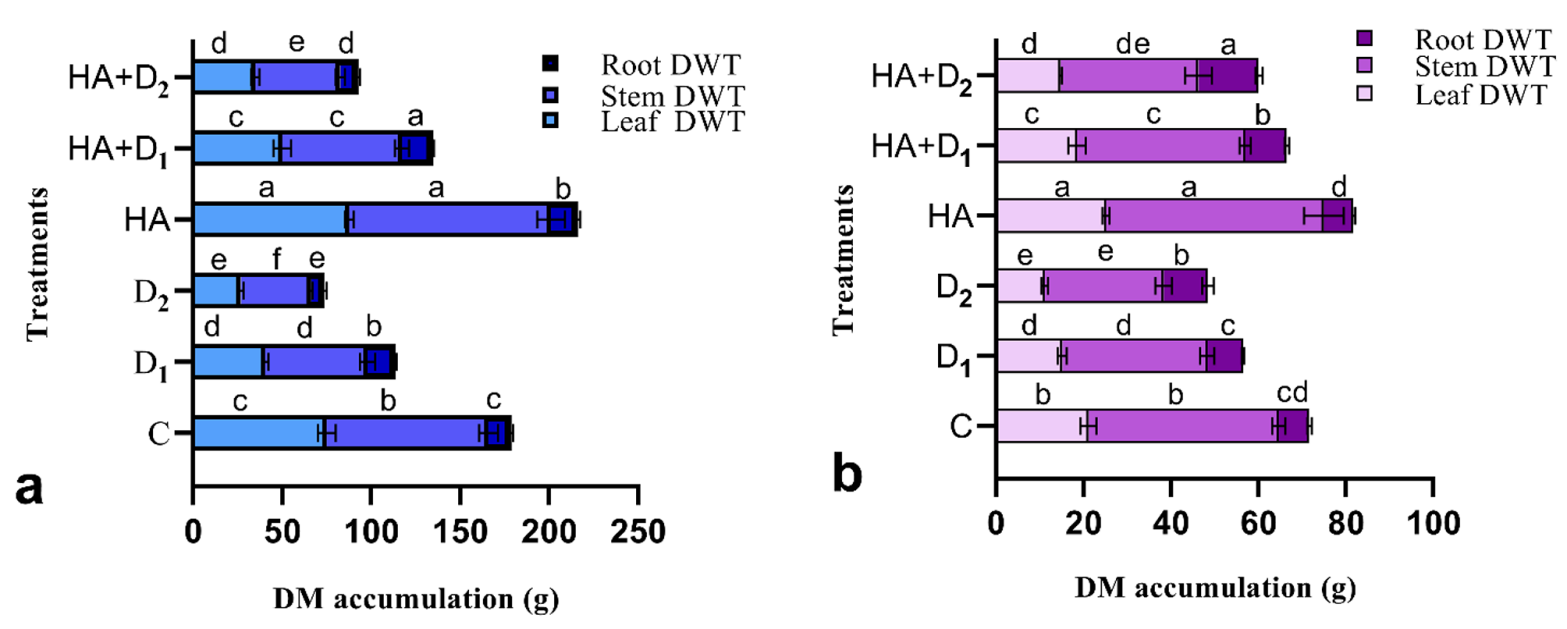
Influence of drought stress and HA on biomass accumulation in (**a**) maize and (**b**) sorghum plants. Shown means ± SD (*n* = 3). Means followed by different letters indicate significant responses whereas those followed by the same letters depict non-significant responses for the respective parameters at Fisher’s test (*P* ≤ 0.05). C; Control, D_1_; drought level 1, D_2_; drought level 2, HA; humic acid

Figure 1 shows the variation in dry matter accumulation in various organs of maize and sorghum plants under different levels of drought stress in the absence and presence of HA priming. Overall, biomass accumulation in leaves and stems decreased as the level of drought stress increased. Compared to the well-watered plants, the D_2_ maize plants lost ~ 64.2% and 56.6% of their foliar and stem biomass, respectively whereas the corresponding D_1_-induced reduction in both foliar and stem biomass were 46.0% and 37.9%. In sorghum, the D_2_ treatment resulted in reductions of 47.2% in leaf biomass and 37.6% in stem biomass compared to 28.4% and 23.8% in these two parameters, respectively under the D_1_ treatment. Regarding roots, the D_1_ maize plants accumulated higher biomass in their roots than their control plants; however, the biomass accumulation in the D_2_ maize plants was substantially lower than the control plants. In sorghum, both the D_1_ and D_2_ treatments significantly (*p* ≤ 0.05) increased root biomass accumulation compared to the well-watered plants. In both plant species, leaves were the most sensitive plant organ to drought stress. HA preconditioning caused general and significant increases in biomass accumulation in all plant organs of both species compared to the control plants. Under drought stress, HA significantly (*p* ≤ 0.05) increased dry matter accumulation compared to the stressed and unprimed plants. The HA-induced increments in dry matter accumulation in leaves, stem, and root were more prominent under D_2_ than D_1_. The increments in these plant organs approached 29.8, 21.1, and 34.8% in maize and 32.8, 16.0, and 36.1% in sorghum, respectively under D_2_ compared to 23.5, 26.4, and 10.5% in maize and 22.0, 16.1, and 16.3% in sorghum, respectively under D_1_.

### Effects of HA on photosynthetic pigments in maize and sorghum plants under drought stress

**Fig. 2:**
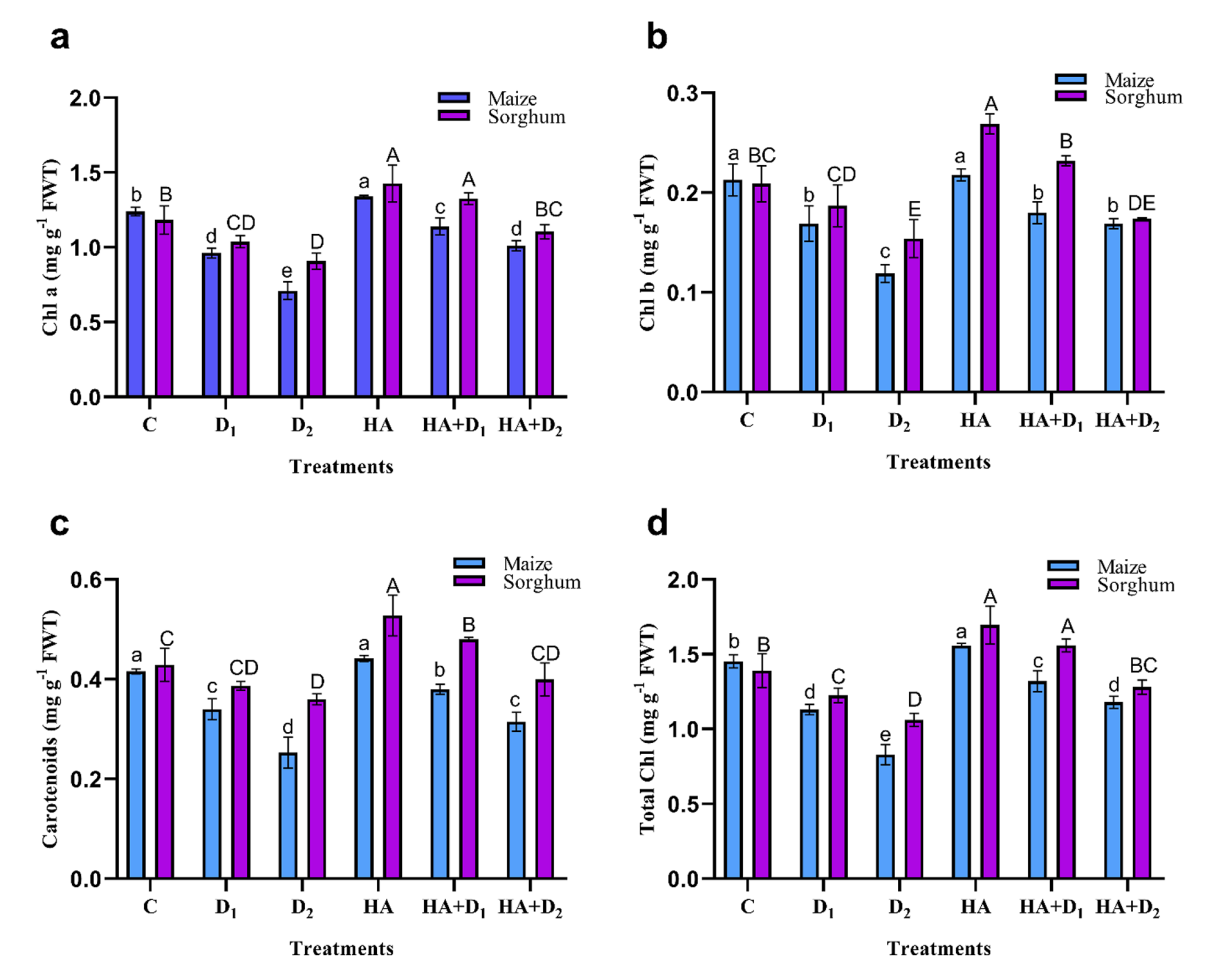
Influence of drought stress and HA on photosynthetic pigments in maize and sorghum plants. Panels are: (**a**) Chl a, (**b**) Chl b, (**c**) Carotenoids, and (**d**) Total chlorophylls. Shown means ± SD (*n* = 3). Means followed by different letters indicate significant responses whereas those followed by the same letters depict non-significant responses for the respective parameters at Fisher’s test (*P* ≤ 0.05) (uppercase letters for sorghum and lowercase letters for maize). C; Control, D_1_; drought level 1, D_2_; drought level 2, HA; humic acid

Drought stress effectively diminished the content of Chl a, Chl b, total chlorophyll, and carotenoids pigments in maize and sorghum leaves compared to those of the well-irrigated plants (Fig. 2). Such a decline was more detectable in the D_2_-maize and sorghum plants than the D_1_ plants. Relative to the control plants, the D_2_-induced reduction in Chl a, Chl b, carotenoids, and total chlorophylls were 42.7, 44.0, 39.3, and 42.9%, respectively, in maize compared to 23.3, 26.0, 16.0, and 23.7% in sorghum. These records were reduced by half under the D_1_ conditions. Maize plants were consistently more sensitive to drought stress than sorghum plants. Under the well-irrigated conditions, HA priming resulted in significant rises (*p* ≤ 0.05) in all photosynthetic pigments in maize and sorghum plants relative to the HA unprimed controls. Under water shortage, the application of HA decreased the drought-triggered reductions in the tested photosynthetic pigments in both plant species. Compared to the D_2_ unprimed maize plants, HA triggered increases of 42.3, 41.6, 24.5, and 42.2% in Chl a, Chl b, carotenoids, and total Chl, respectively. Significantly lower HA-induced responses (*p* ≤ 0.05) were observed in the D_1_ primed maize plants. In sorghum, compared to the D_1_ unprimed controls, the D_1_ primed plants accumulated 27.8, 24.4, 24.1, and 27.3%, higher Chl a, Chl b, carotenoids, and total Chl, respectively. Interestingly, the responses under the D_2_ primed sorghum were significantly lower than the D_1_ primed sorghum plants.

### Effects of HA on photosynthetic CO_2_ assimilation in maize and sorghum plants under drought stress

**Fig. 3:**
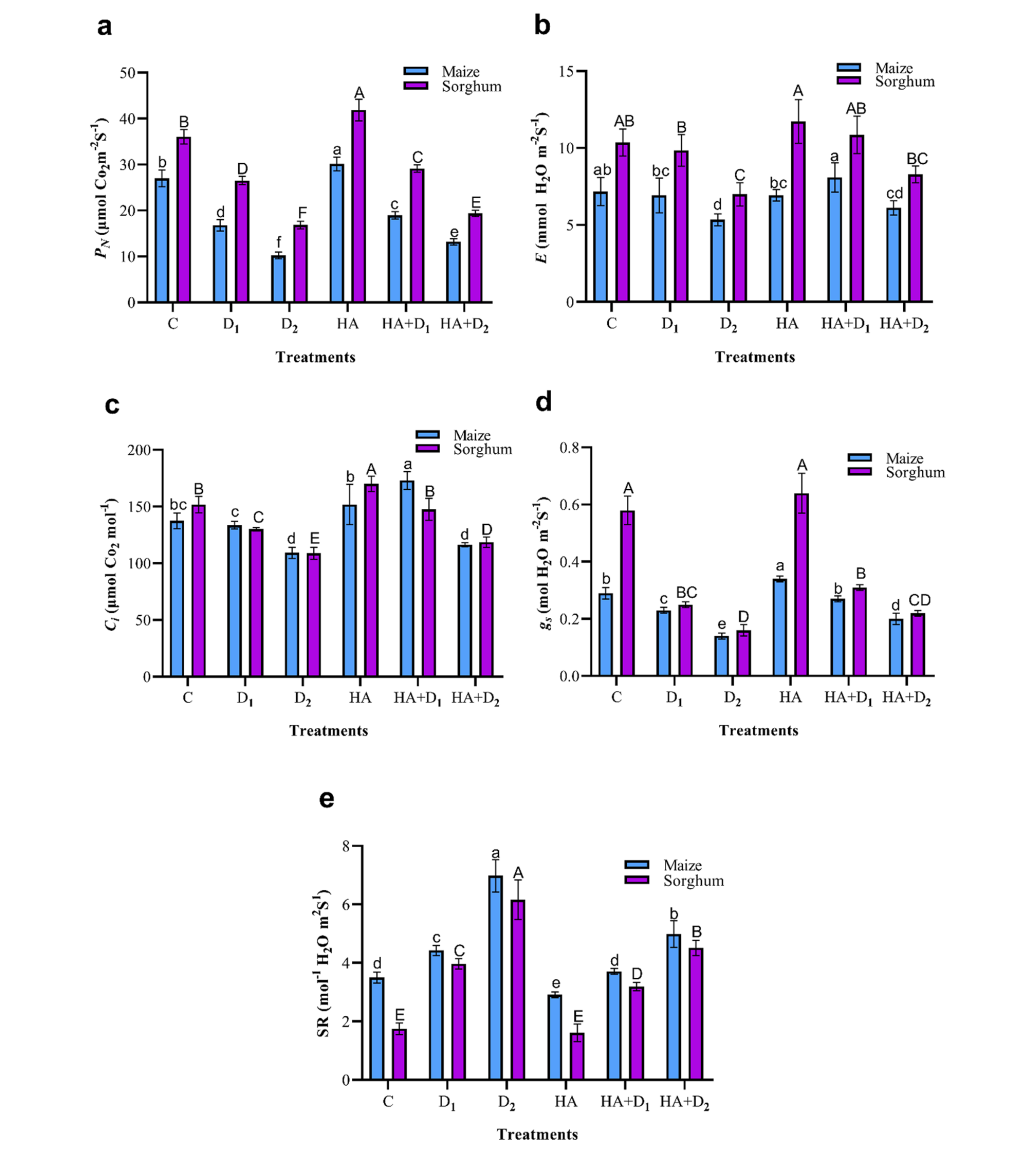
Influence of drought stress and HA on photosynthetic gas exchange in maize and sorghum plants. Panels are: (**a**) photosynthetic rate (*P*_*N*_), (**b**) transpiration rate (*E*), (**c**) internal CO_2_ (*Ci*), (**d**) stomatal conductance (*g*_*s*_), and (**e**) stomatal resistance (SR). Shown means ± SD (*n* = 3). Means followed by different letters indicate significant responses whereas those followed by the same letters depict non-significant responses for the respective parameters at Fisher’s test (*P* ≤ 0.05) (uppercase letters for sorghum and lowercase letters for maize). C; Control, D_1_; drought level 1, D_2_; drought level 2, HA; humic acid

In addition to the photosynthetic pigments, CO_2_ assimilation and its related parameters are vital metrics to evaluate the impact of drought stress and HA as stress-alleviating biostimulant on photosynthesis in maize and sorghum plants. As shown in Fig. 3, both the D_1_ and D_2_ water deficiency levels significantly (*p* ≤ 0.05) reduced photosynthetic CO_2_ assimilation parameters, including photosynthetic rate (*P*_*N*_), transpiration rate (*E*), internal CO_2_ concentration (C_*i*_), and stomatal conductance (g_s_) in leaves of maize and sorghum plants, albeit at significantly different magnitudes. However, the two drought treatments significantly (*p* ≤ 0.05) increased stomatal resistance (SR) in both plant species. Compared to the well-watered plants, the D_2_ treatment caused reductions of 62.1, 20.5, 25.5, and 49.7% in *P*_*N*,_ C_*i*_, *E*, and g_s_ in maize. In sorghum, the corresponding D_2_-induced reductions in these parameters were 53.3, 28.3, 32.5, and 71.5%, respectively. In both plant species, D_1_ reduced these four parameters, however to a significantly lower extent compared to D_2_. HA significantly augmented photosynthetic indices in both plant species under adequate water supply. Furthermore, HA priming mitigated stress-deleterious changes in gas-exchange characteristics compared to the stressed unprimed plants of both species. Compared to the stressed unprimed maize plants, HA caused increments of 12.7, 29.5, and 19.5% in *P*_N_, C_*i*_, and g_s_, respectively, under D_1_ whereas the corresponding values in these parameters under D_2_ approached 29.2, 6.6, and 40.3%. In sorghum, the HA-induced enhancement in these three parameters under D_1_ was 9.9, 13.5, and 24.6% compared to 15.0, 9.0, and 35.4% respectively, under D_2_. On the other hand, HA priming had a non-significant effect (*p* ≤ 0.05) on *E* whereas it significantly decreased SR under both drought levels in the two plant species.

### Effects of HA on carbohydrate, TSP, and proline in maize and sorghum plants under drought stress

**Fig. 4:**
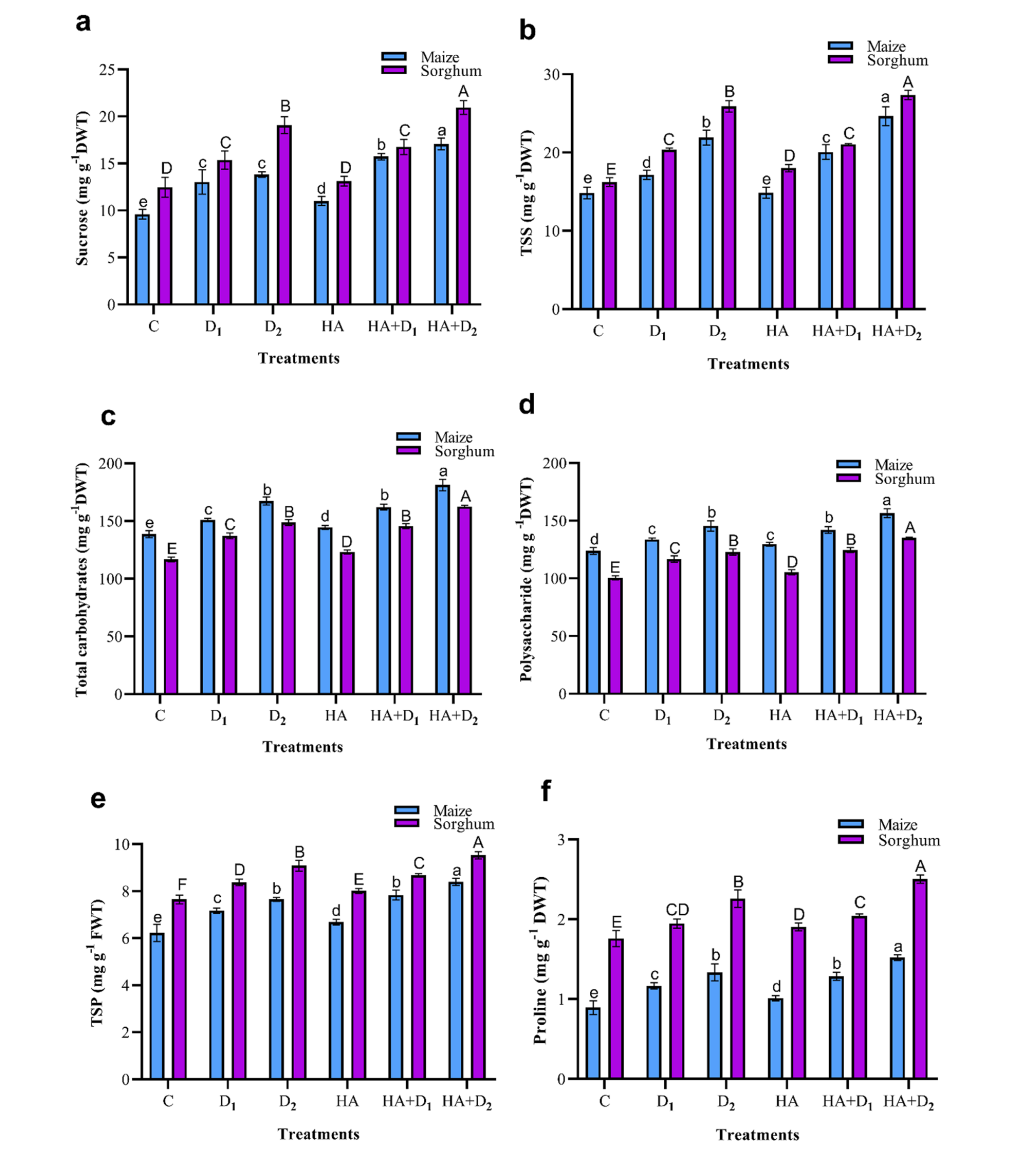
Influence of drought stress and HA on osmolytes content in maize and sorghum plants. Panels are: (**a**) sucrose, (**b**) total soluble sugars (TSS), (**c**) total carbohydrates, (**d**) polysaccharides, (**e**) total soluble proteins (TSP), and (**f**) proline. Shown means ± SD (*n* = 3). Means followed by different letters indicate significant responses whereas those followed by the same letters depict non-significant responses for the respective parameters at Fisher’s test (*P* ≤ 0.05) (uppercase letters for sorghum and lowercase letters for maize). C; Control, D_1_; drought level 1, D_2_; drought level 2, HA; humic acid

Relative to the well-watered plants, drought stress triggered significant increments in the cellular content of sucrose, TSS, total carbohydrates, and polysaccharides in maize and sorghum plants (Fig. 4a-d). Relative to the well-watered plants, the D_2_ maize plants accumulated 43.9, 47.7, 20.7, and 17.5% higher sucrose, TSS, total carbohydrates, and polysaccharides, respectively. In the D_2_ sorghum plants, the corresponding values of these carbohydrate fractions were 53.0, 59.4, 27.4, and 22.2%, respectively. D_1_ also induced significant accumulation (*p* ≤ 0.05) of these carbohydrates resides albeit to a significantly lower extent in both species. Under the well-watered conditions, seed priming with HA either induced slight or non-significant changes in most of the tested carbohydrate residues in both plant species. However, under water limitation, HA priming increased the levels of the tested carbohydrate residues compared to the stressed unprimed controls with stronger responses under D_2_ than D_1_. Indeed, the D_2_ primed plants had the highest level of carbohydrates among treatments in both plant species with 23.5, 12.5, 8.3, and 7.7% increases in sucrose, TSS, total carbohydrates, and polysaccharides, respectively compared to their D_2_ and unprimed maize plants. In sorghum, the corresponding magnitudes of the HA induction in the above carbohydrate resides approached 9.8, 5.7, 9.2, and 9.9%, respectively. Regardless of treatments, sorghum plants maintained consistently higher sucrose and TSS than maize plants, whereas the latter had consistent superiority over sorghum in total carbohydrates and polysaccharides.

Maize and sorghum plants exhibited pronounced increases in TSP and proline contents in response to different levels of drought stress (Fig. 4e, f). Relative to the well-irrigated plants, the D_2_ maize and sorghum plants accumulated 22.9% and 18.7% higher TSP compared to 49.6%, and 28.4% higher proline, respectively. Regardless of treatments, sorghum plants had consistently higher levels of TSP and proline than maize plants. Interestingly, sorghum leaves accumulated more than two-folds higher proline than maize. The contents of both TSP and proline were also remarkably stimulated by HA in both species under both stress and non-stress circumstances. Under the well-watered conditions, HA significantly (*p* ≤ 0.05) increased TSP and proline in maize and sorghum plants. The HA-induced accumulation of these analytes increased as drought stress increased. Compared to the D_2_ unprimed plants, the HA-induced TSP and proline accumulation in maize were 9.7% and 14.1%, respectively. In sorghum, the HA-induced TSP and proline were 4.9% and 10.9%, respectively. Under D_1_, the HA-induced changes were significantly (*p* ≤ 0.05) lower than those induced by D_2_, yet they were significantly higher than their D_1_ unprimed peers.

### Effects of HA on oxidative stress markers in maize and sorghum plants under drought stress

**Fig. 5:**
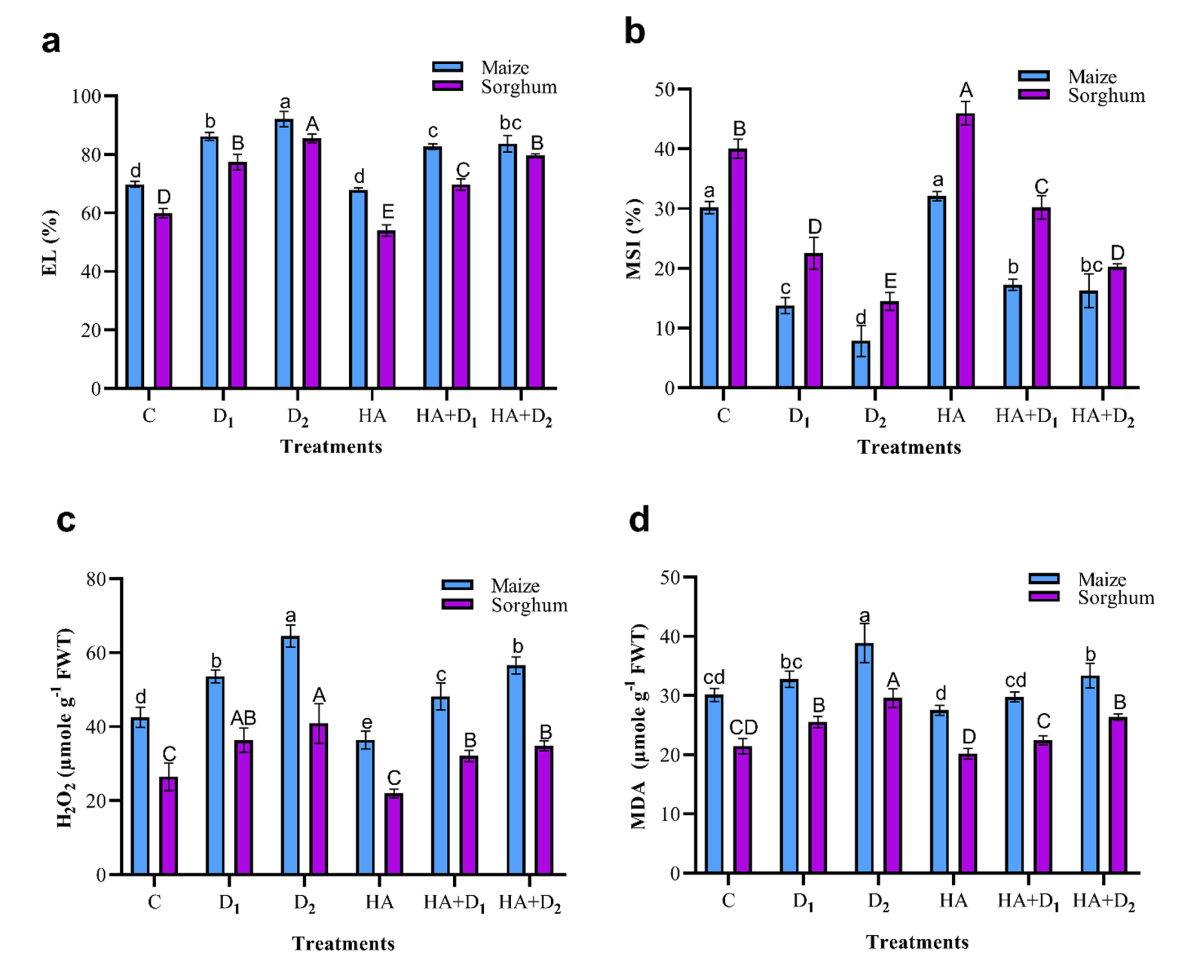
Influence of drought stress and HA on oxidative stress markers in maize and sorghum plants. Panels are: (**a**) electrolyte leakage (EL), (**b**) membrane stability index (MSI), (**c**) hydrogen peroxide (H_2_O_2_), and (**d**) malondialdehyde (MDA). Shown means ± SD (*n* = 3). Means followed by different letters indicate significant responses whereas those followed by the same letters depict non-significant responses for the respective parameters at Fisher’s test (*P* ≤ 0.05) (uppercase letters for sorghum and lowercase letters for maize). C; Control, D_1_; drought level 1, D_2_; drought level 2, HA; humic acid

Decreasing soil water content led to a substantial rise in H_2_O_2_ production, MDA, and EL in maize and sorghum plants (Fig. 5). Relative to the well-watered plants, the D_2_ conditions caused increments of 51.5, 29.1, and 31.9% in H_2_O_2_, MDA, and EL in maize, respectively. In sorghum, the corresponding values of these biomarkers were 54.5, 38.2, and 42.6%, respectively. The D_1_-induced rise in the above biomarkers was much lower in both plants. In contrast, drought reduced the MSI in maize and sorghum, compared to the well-watered plants. The D_2_ conditions decreased MSI by 73.9% in maize and 63.8% in sorghum. Seed priming in HA either slightly lowered or did not statistically change these biomarkers compared to the well-watered plants. It also increased the MSI, particularly in sorghum plants. Compared to the D_2_ unprimed maize plants, HA reduced the levels of H_2_O_2_, MDA, and EL by 12.3, 14.2, and 9.1%, respectively. Relative to the D_1_ unprimed maize plants, the HA-induced reduction in the above markers approached 10.1, 9.2, and 4.0%, respectively. In the D_2_ primed sorghum plants, the corresponding values of the HA-triggered decline were 14.8, 10.8, and 6.8% in H_2_O_2_, MDA, and EL compared to their corresponding D_2_ unprimed peers. Compared to the D_1_ unprimed sorghum plants, HA treatment caused a decline of 11.7, 12.1, and 9.9% in H_2_O_2,_ MDA, and EL, respectively.

### Effects of HA on the activity of antioxidant system in maize and sorghum plants under drought stress

**Fig. 6:**
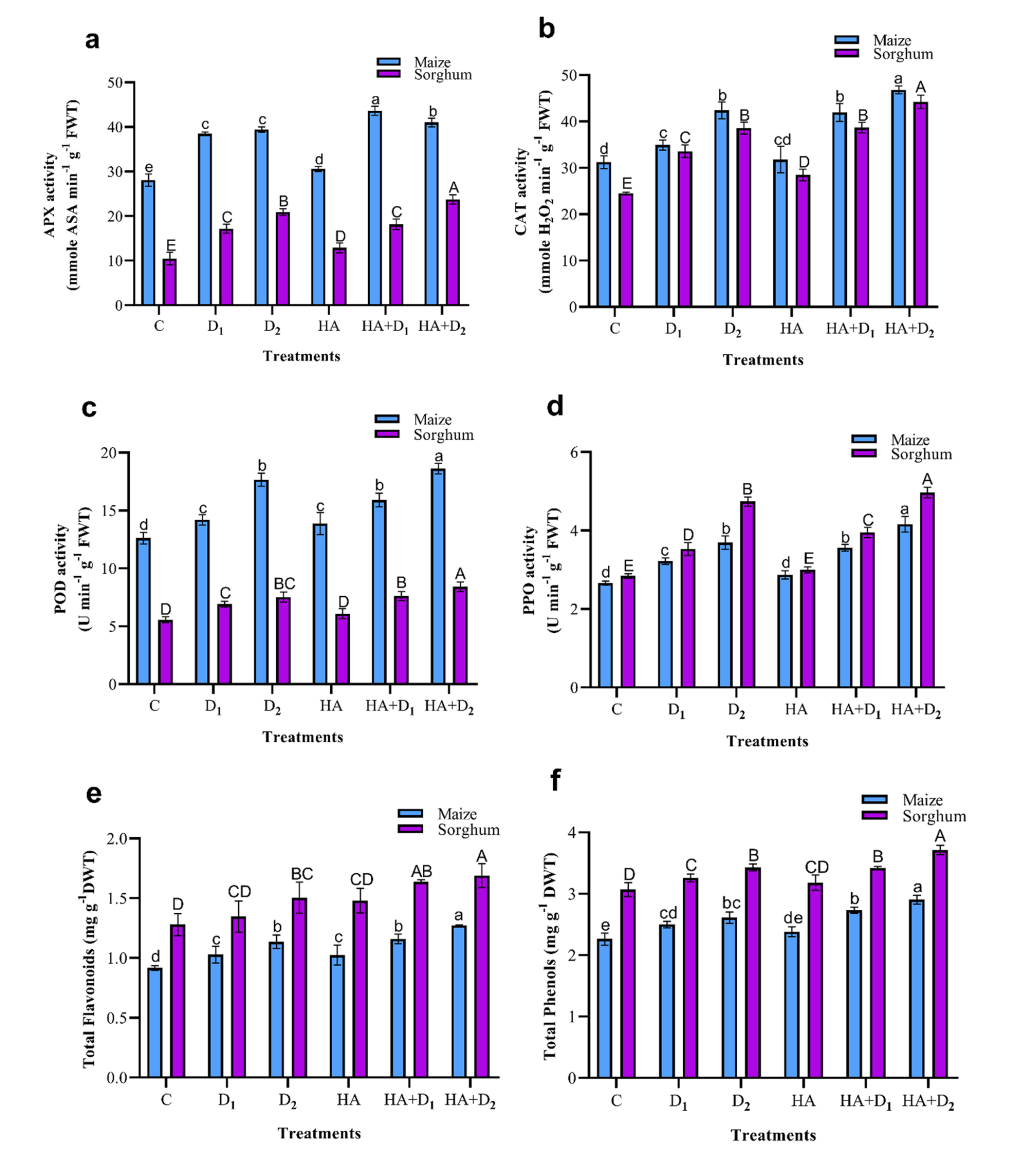
Influence of drought stress and HA on enzymic and non-enzymic antioxidants in maize and sorghum plants. Panels are: (**a**) ascorbate peroxidase (APX), (**b**) catalase (CAT), (**c**) peroxidase (POD), (**d**) polyphenol oxidase (PPO), (**e**) total flavonoids, and (**f**) total phenols. Shown means ± SD (*n* = 3). Means followed by different letters indicate significant responses whereas those followed by the same letters depict non-significant responses for the respective parameters at Fisher’s test (*P* ≤ 0.05) (uppercase letters for sorghum and lowercase letters for maize). C; Control, D_1_; drought level 1, D_2_; drought level 2, HA; humic acid

Under the well-watered plants, maize had higher natural activities of APX, CAT, and POD than sorghum, whereas the relation was reversed for PPO (Fig. 6a-d). Compared to the well-watered plants, drought significantly (*p* ≤ 0.05) augmented the activity of APX, CAT, POD, and PPO in the two species, and the responses increased as the intensity of drought stress increased. For instance, D_1_ induced increments of 36.9, 11.9, 12.5, and 21.3% in the activities of APX, CAT, POD, and PPO in maize whereas the corresponding values in these enzymes were 64.0, 37.0, 24.3, and 24.0% in sorghum. The D_2_-triggered responses in these enzymes were much higher than D_1_ with increments of 40.3, 35.8, 39.9, and 39.0% in maize compared to 99.8, 57.3, 35.1, and 67.1% in sorghum. HA priming showed either slight positive or non-significant effects (*p* ≤ 0.05) on the activity of the tested antioxidant enzymes under the adequate water input. Under water shortage, HA generally increased the activity of the tested enzymes in both species compared to the corresponding controls with the exclusion of APX in D_1_ sorghum plants which showed a non-significant response. Compared to the D_2_ unprimed plants, the HA-elicited differential responses in the tested enzymes in maize and sorghum, respectively were CAT (10.5% and 14.7%), POD (5.4% and 12.0%), and PPO (12.6% and 4.7%). Relatively similar responses were observed under D_1_ conditions in both sorghum and maize.

Unlike antioxidant enzymes, analysis of the well-watered plants indicated that sorghum has higher natural levels of total flavonoids and phenols than maize (Fig. 6e, f). Compared to the well-watered plants, drought stress resulted in remarkable increases in the content of flavonoids and phenolics in the two species, and the response was much higher under D_2_ than D_1_. Compared to the well-watered plants, the D_2_ maize leaves had 23.8% and 15.4% higher flavonoids and phenolic, respectively whereas the corresponding values of these two secondary metabolites were 17.5% and 11.8% in sorghum. The D_1_-induced accumulation in flavonoids and phenolics was significantly (*p* ≤ 0.05) lower than D_2_. Further, HA treatment showed a modest increase in the level of flavonoids and phenols in both species under adequate watering conditions. Compared to the D_1_ unprimed plants, HA significantly (*p* ≤ 0.05) increased the content of flavonoids and phenols by 12.7% and 9.3% in maize relative to 21.6% and 4.9% in sorghum, respectively. Relative to the D_2_ unprimed plants, the HA-induced increases in the above non-enzymatic antioxidants approached 12.0% and 11.2% in maize compared to 12.3% and 8.2% in sorghum, respectively.

### Correlation studies and PCA analysis

**Fig. 7:**
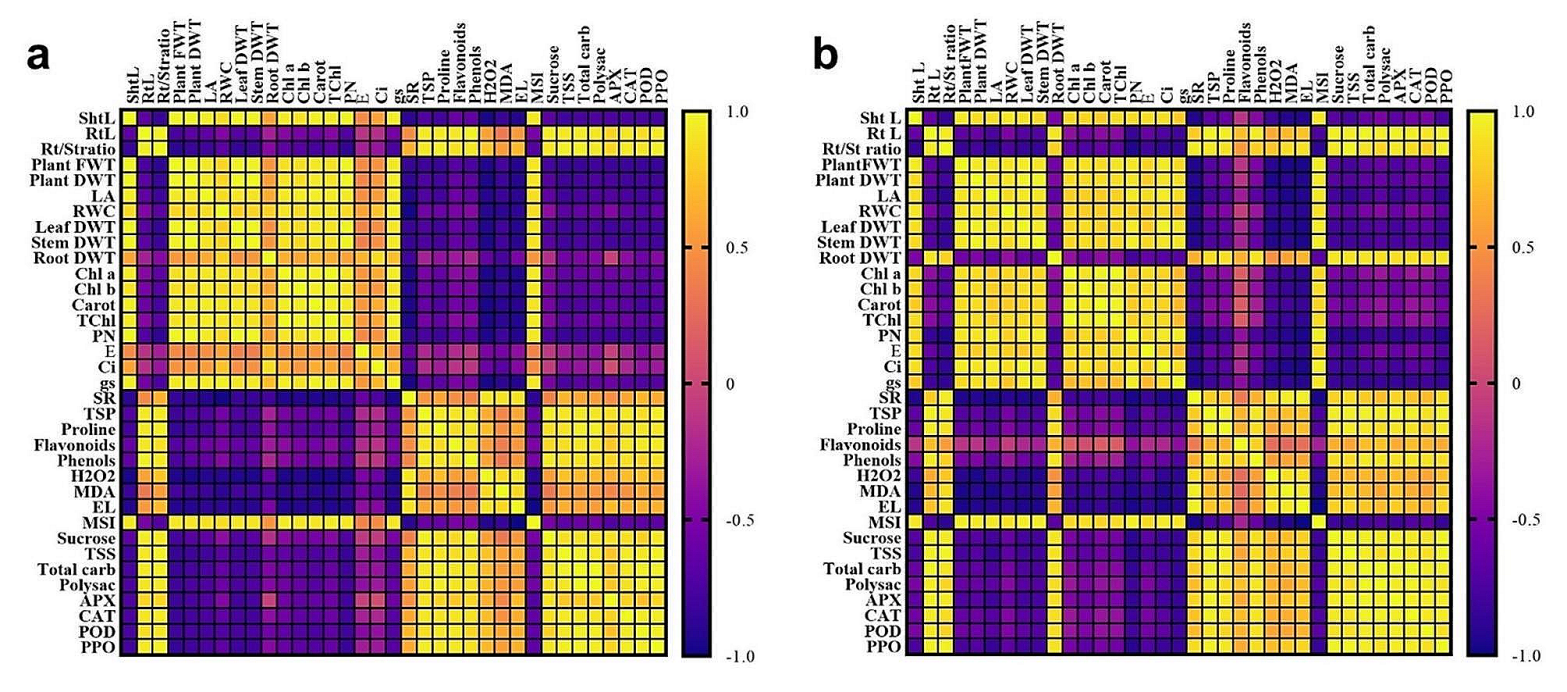
Pearson’s correlation analysis between all the tested parameters in (**a**) maize and (**b**) sorghum plants. Sht L; shoot length, Rt L; root length, Rt/Sht ratio; root/shoot ratio, LA; leaf area, RWC; relative water content, Chl a; chlorophyll a, Chl b; chlorophyll b, Carot; carotenoids, T Chl; total chlorophyll, *P*_*N*_; photosynthetic rate, *E*; transpiration rate, *C*_*i*_; internal CO_2_, *g*_*s*_; stomatal conductance, SR; stomatal resistance, TSS; total soluble sugars, Total carb; total carbohydrates, Polysac; polysaccharides, TSP; total soluble protein, H_2_O_2_; hydrogen peroxide, MDA; malondialdehyde, EL; electrolyte leakage, MSI; membrane stability index, APX; ascorbate peroxidase, CAT; catalase, POD; peroxidase, and PPO; polyphenol oxidase

Pearson’s correlation metrics were performed to explore the degree of association among the tested morphological, physiological, and biochemical responses of maize and sorghum plants (Fig. 7) across different treatments. In both species, growth indices (shoot length, plant fresh and dry weights, and leaf area), RWC, dry matter accumulation (leaf and stem), photosynthetic pigments (Chl a, Chl b, Carot, and T Chl), photosynthetic gas exchange (*P*_*N*_, *E, C*_*i*_, and *g*_*s*_), and MSI showed positive strong correlations with each other. In contrast, the responses of these traits exhibited negative strong correlations with root length, root/shoot ratio, and root dry weight (particularly in sorghum), TSP, proline, carbohydrate fractions, H_2_O_2_, MDA, EL, flavonoids, phenols, and antioxidant enzymes. On the other hand, the latter traits maintained strong positive correlations with each other in both maize and sorghum plants.

**Fig. 8:**
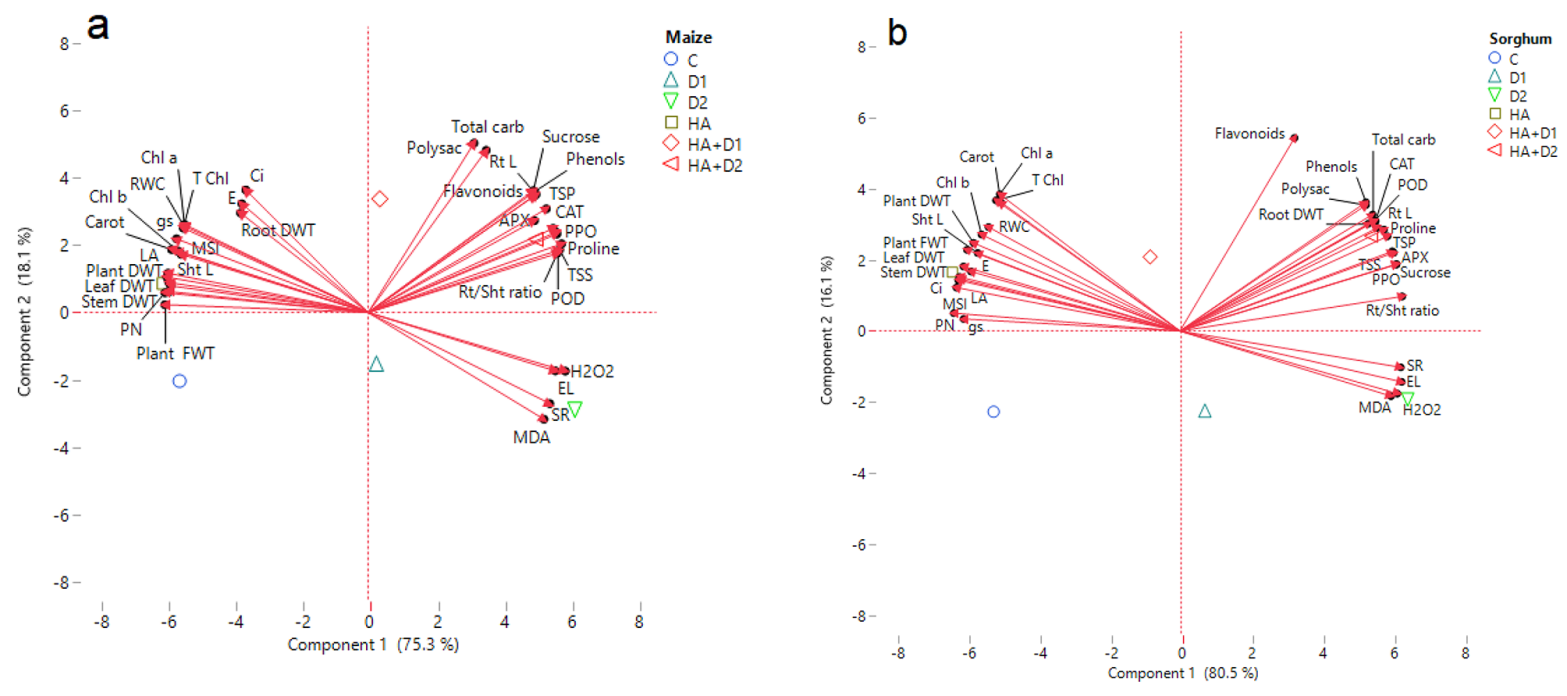
PCA biplot of growth, physiological, and biochemical traits in (**a**) maize and (**b**) sorghum plants. Sht L; shoot length, Rt L; root length, Rt/Sht ratio; root/shoot ratio, LA; leaf area, RWC; relative water content, Chl a; chlorophyll a, Chl b; chlorophyll b, Carot; carotenoids, T Chl; total chlorophyll, *P*_*N*_; photosynthetic rate, *E*; transpiration rate, *C*_*i*_; internal CO_2_, *g*_*s*_; stomatal conductance, SR; stomatal resistance, TSS; total soluble sugars, Total carb; total carbohydrates, Polysac; polysaccharides, TSP; total soluble protein, H_2_O_2_; hydrogen peroxide, MDA; malondialdehyde, EL; electrolyte leakage, MSI; membrane stability index, APX; ascorbate peroxidase, CAT; catalase, POD; peroxidase, and PPO; polyphenol oxidase

To summarize the responses of maize and sorghum plants to the tested different treatments, PCA as a type of multivariate statistical analysis was applied (Fig. 8). The results revealed that the two principal components (PC1 and PC2) showed a variance of 93.4% in maize and 96.6% in sorghum of total data variability. In both plant species, three distinct clusters were obtained in the biplot: the first cluster included oxidative markers (H_2_O_2_, MDA, and EL), and SR clustered together and were strongly linked to D_2_ stress. The second cluster included growth indices (shoot length, plant fresh and dry weights, and leaf area), RWC, dry matter accumulation (leaf, stem, and root), photosynthetic pigments (Chl a, Chl b, Carot, and T Chl), photosynthetic gas exchange (*P*_*N*_, *E, C*_*i*_, and *g*_*s*_), and MSI. These parameters were grouped under HA treatment supporting its potential role in improving maize and sorghum growth and photosynthesis under well-watered conditions. The third cluster includes root dry weight (mainly in sorghum), as well as root length (RL), root/shoot ratio (R/S), antioxidant enzymes (APX, CAT, POD, and PPO), flavonoids, phenols, carbohydrate fractions, TSP, and proline in both plant species. These parameters showed high association with HA + D_2_ treatment reflecting the possible effectiveness of HA in alleviating drought stress in both studied species.

## Discussion

Water limitation is a major abiotic stress that severely inhibits plant growth via the induction of numerous morphological, physiological, and biochemical alterations [14, 61, 62]. Drought-induced morphological alterations are the key signs of environmental stress on plants [63]. Our current results revealed significant retardation in the tested growth characteristics, including shoot length, plant fresh and dry weight, leaf area, and dry matter accumulation in the stem and leaves of maize and sorghum plants in response to the tested levels of drought stress. In addition, the drought-induced repression in the growth and its related traits increased as drought levels increased (Table 1; Fig. 1). These results are consistent with the reported reductions in the growth and its related traits in pot-grown maize seedlings [64] and field-grown sorghum seedlings [65] under limited water availability. Such defects are mainly attributed to the drought-induced perturbation in water absorption, which increases cell dehydration and inhibits cell division, expansion, and proliferation [14]. Unlike the above drought-triggered suppression in growth, our results revealed significant improvement of root traits including length, dry matter, and root/shoot ratio in maize and sorghum plants in response to the applied levels of water limitation (Table 1; Fig. 1). These results indicate that the stressed maize and sorghum plants invest in their root systems via allocation of more resources to their roots to support the changes in root morphology necessary to maximize water and nutrient uptake under drought stress [14]. Consistent with that, it has been reported that droughted green-house maize and sorghum plants develop deeper roots to absorb enough water and reduce the incidence of water deficits [66, 67]. Further, it has been reported that maize hybrids that develop higher root/shoot ratio values can effectively survive drought stress [68]. Our findings are thus in harmony with Shoaib et al. [11], who reported that drought-stressed plants prioritize resource allocation to the root system, resulting in greater root length, biomass, and root/shoot ratio, allowing plants to absorb more water. Our results also agree with the coordinated resource allocation hypothesis which suggests that plants usually drive their coordinated resource allocation toward the plant organs that are essential in mitigating the stress they encounter [69].

HA priming improved maize and sorghum growth under well-watered conditions and significantly alleviated drought adversities in both plant species via enhancing growth characteristics and biomass accumulation in plant leaves, stem, and root (Table 1; Fig. 1). Similar HA-induced improvements in growth indices have been reported in hydroponically grown maize seedlings [70], greenhouse-grown sorghum at reproductive stage [71], and other plant species including rice [32], wheat [72], and soybean [73]. The positive effects of HA on the growth of different plant organs support its pleiotropic effects on physiological and biochemical signaling pathways that mediate drought resilience [30, 74, 75]. Such HA-drought alleviating potential might be ascribed to its role in the regulation of endogenous hormonal balance, as it down-regulates ABA biosynthesis-related genes while upregulates IAA-related genes, resulting in enhancing photosynthetic activities and plant growth [76]. In addition, humic substances exert promotive effects on cell membrane permeability and thus enhance the uptake of water and nutrients; responses that eventually improve plant growth and drought tolerance [77]. Further, the effective role of HA in cell division, which is linked to increased chlorophyll and antioxidants has been reported [26]. Therefore, these results support the dual potential of HA priming as an effective strategy to improve plant growth under adequate and limited irrigation conditions.

RWC reflects the plant’s ability to uptake water from the soil and acts as a measure of plant resistance against drought [78]. In the current study, water limitation disturbed the water balance and caused a remarkable decline in leaf RWC in maize and sorghum plants (Table 1). These findings coincide with recent reports [23]. The suppressive effects of drought stress on RWC might be ascribed to its negative impact on the expression of genes involved in the signal transduction and modulation of proteins mediating channels and transporters involved in water movement in plants [79]. HA has markedly compensated for the drought-induced disorders in leaf RWC in maize and sorghum (Table 1). Such HA-promoting effects have been reported under various stress conditions, including drought and salinity [80–82]. A similar promotive role of HA has been described in HA-treated millet [26] and maize seedlings and plants grown in greenhouse and hydroponic systems [30, 76]. The stimulatory effect of HA on leaf water content may be due to its positive contribution to membrane and organelles stabilization, and cell growth maintenance.

Reductions in the main photosynthetic pigments and photosynthetic gas exchange parameters are critical consequences of water deficit [83, 84]. In this investigation, water limitation substantially declined Chl a, Chl b, total Chl, and carotenoids, with more pronounced adverse effects in maize than in sorghum plants (Fig. 2). Similar reductions in chlorophyll and carotenoid pigments had previously been reported in maize and sorghum plants under polyethylene glycol-induced drought stress [23]. These results are attributed to the drought-triggered degradation of chlorophyll and carotenoid, inhibition of activity and synthesis of chloroplast proteins, and reductions in chlorophyll precursors [85, 86]. It is worth noting that carotenoids were less affected by drought than chlorophyll, particularly in sorghum plants which agrees with its reported role as a drought-resistance strategy due to their photoprotective and antioxidant properties [23]. The reductions in photosynthetic pigments and RWC were associated with parallel reductions in photosynthetic gas exchange parameters such as *P*_*N*_, *E*, *g*_*s*_, and *C*_*i*_ in the stressed maize and sorghum plants (Fig. 3). These results are supported by the reported suppression of photosynthetic components in the droughted maize and sorghum seedlings [64, 87], wheat [84], and rice [88]. Such reductions in photosynthetic capability are related to the stomatal closure, which is a general response of plants under water deficit as a strategy to maintain their water content; however, it substantially reduces photosynthetic rate, leaves transpiration, and mesophyll cells CO_2_ [89, 90]. The drought-triggered reduction in CO_2_ assimilation will substantially reduce photoassimilates production and allocation to plant organs [86, 91]. It also triggers significant perturbations in nutrient and water homeostasis, hydraulic conductivity, and reduces cell division and enlargement, leaf growth, and root proliferation [92]. Altogether, these responses explain the observed drought-induced retardation in growth indexes of both maize and sorghum reported in the current study (Table 1; Fig. 1). HA boosted photosynthetic pigments, CO_2_ assimilation and its related parameters in the well-watered and stressed maize and sorghum plants. Similar HA-ameliorative effects have been reported in the photosynthetic machinery of many plants such as rapeseed [93], maize seedlings growing in hydroponics [30], millet [26], and sugar beet [94]. The positive role of HA in alleviating drought-elicited decreases in photosynthetic rate, stomatal conductance, and transpiration may be attributed to its positive interference with regulation of stomatal opening via the activation of plasma membrane H^+^-ATPase [95]. This hypothesis is supported by the reported enormous induction of photosynthetic rate and transpiration and their associated increase in stomatal conductance by HA in sugarcane plants [96]. Such HA-stimulatory effects on photosynthetic pigments and photosynthetic rate reveal its positive role in enhancing photosynthetic efficiency and explain the HA-induced improvement in growth, dry matter accumulation, and overall drought tolerance in both maize and sorghum in our study and others [76].

Oxidative stress is a common consequence of the drought-induced overproduction of ROS in plants [97]. Compared to the well-watered plants, water deficit considerably increased the accumulation of the oxidative stress markers such as H_2_O_2_ and MDA as well as the leaf EL with significantly higher records in maize than sorghum (Fig. 5). Similar induction of oxidative stress biomarkers under limited water supply have been reported in alpine [98], maize and sorghum seedlings in solution cultures [23] as well as in pot-grown sorghum at reproductive growth [99]. The drought-induced accumulation of H_2_O_2,_ along with other ROS, activates a series of consecutive events including membrane lipid peroxidation and MDA accumulation, reduction of membrane integrity, and induction of membrane electrolyte leakage. Interestingly, our heatmap correlation analysis (Fig. 7) and PCA biplot (Fig. 8) supported these findings and revealed a strong positive correlation among H_2_O_2_, MDA, EL, and SR in the stressed maize and sorghum plants. The significantly lower oxidative stress markers in sorghum, compared to maize under normal conditions and drought stress (Fig. 5) suggest that sorghum might have developed more effective strategies for scavenging ROS produced from physiological processes under the well-irrigated and drought conditions than maize. It also agrees with the reported higher natural drought tolerance in sorghum than maize. HA pretreatment effectively decreased the level of H_2_O_2_, MDA, and EL; responses that significantly reduced the oxidative stress in maize and sorghum under the current experimental conditions. Such HA-suppressive role on oxidative stress has been reported in millet [26], rice [28, 31], and rapeseed [100]. These responses are attributed to the reported ability of HA to restore cell redox potential through scavenging H_2_O_2_ and consequently decreasing MDA and EL [80, 81]. Such ameliorative effects of HA alleviated the drought-induced oxidative damage in the plasma membrane and significantly increased the MSI in both species (Fig. 5). Altogether, these responses explain the observed HA-induced improvement in the growth of maize and sorghum plants under water limitation.

To mitigate drought-induced cellular water deficiency and oxidative damage, and to maintain the water osmotic homeostasis, plants employ common and species-specific physiological responses such as osmotic adjustment and activation of their antioxidant systems [76]. Our results indicated that both maize and sorghum accumulated significantly high levels of osmoprotectants (sucrose, TSS, total carbohydrates, polysaccharide, TSP, and proline; Fig. 4), antioxidant enzymes (APX, CAT, POD, and PPO; Fig. 6a-d), and non-enzymatic secondary metabolites (flavonoids and phenolics; Fig. 6e, f) in response to increased water limitation. Interestingly, sorghum plants outperformed maize plants in synthesizing the tested osmoprotectants (sucrose, TSS, TSP, and proline) and non-enzymic antioxidants (flavonoids and phenolics) whereas maize surpassed sorghum in the foliar activity of antioxidant enzymes, particularly APX, CAT, and POD (Fig. 6a-c). Similar drought-induced accumulation of the above osmoprotectants has been reported in rapeseed [101], wheat [13], greenhouse-grown maize seedlings and sorghum plants [12, 102]. Also, the drought-induced activation of antioxidant system-related metabolites and enzymes has been described in tea [103], tomato [104], and greenhouse-grown maize and sorghum plants [10, 105]. Our heatmap correlation analysis supported these findings and revealed positive and strong correlations between oxidative stress markers (H_2_O_2_, MDA, and EL) and the tested osmolytes (carbohydrates, TSP, and proline), non-enzymic secondary metabolites (flavonoids and phenolics), and antioxidant enzymes (APX, CAT, POD, and PPO) (Fig. 7). The above drought-induced accumulation of soluble carbohydrates, TSP, and proline contribute significantly to the regulation of cell osmotic potential, activation of scavenging excess ROS, and overall cellular osmotic homeostasis; responses that eventually enhance water absorption, cell turgidity, photosynthesis, and plant growth [32, 61, 106]. Further, the total flavonoids and phenols accumulated in response to drought serve as effective electron donors and consequently reduce free radicals and protect cells from their oxidative potential [107, 108]. Furthermore, the induced activities of the tested antioxidant enzymes (APX, CAT, and POD) will effectively catalyze the breakdown of H_2_O_2_, reduce its levels in leaves, and protect plants from its induced abnormalities [108]. It is worth mentioning that the differential accumulation of osmoprotectants, non-enzymic, and enzymic antioxidants in maize and sorghum most likely stand behind their differential capabilities in withstanding drought stress. Also, the high level of MDA in maize, compared to sorghum, despite its higher activities of antioxidant enzymes may reflect a more intensive oxidative stress in maize than sorghum. The above three mechanisms (accumulation of osmoprotectants, non-enzymic antioxidants, activation of antioxidant enzymes) are integrated in different ways in maize and sorghum to shape the observed differences in the responses of these two species to drought stress.

HA pretreatment further enhanced the accumulation of the tested osmolytes in the control and the stressed maize and sorghum plants (Fig. 4). Such HA-induced accumulation of osmolytes has also been shown in savory [109] and greenhouse-grown sorghum during seedling and reproductive growth [71, 74]. The PCA biplot supported these findings and revealed a strong association of these osmolytes with HA + D_2_ treatment in maize and sorghum (Fig. 8). The HA-induced accumulation of these osmolytes enhances plant water status, photosynthetic efficiency, and regulates metabolic responses that eventually mitigate the drought-triggered damage and improves overall plant growth [76].

The drought-induced accumulation of non-enzymatic antioxidants and activation of antioxidant enzymes were further increased in response to HA priming in both maize and sorghum (Fig. 6a-f). HA was generally more effective under high level of drought stress (D_2_) than the lower level (D_1_), and these findings are supported by PCA biplot, which showed that enzymatic and non-enzymatic antioxidants were also associated with HA + D_2_ treatment. These findings coincide with the reported capability of HA to boost plant enzymatic and non-enzymatic defense systems for abiotic stress mitigation [110]. A similar HA-induced antioxidant system has been described in millet [26], sugarcane [96], rapeseed [100], and greenhouse-grown and HA-foliar sprayed maize at reproductive stage [111]. Such HA-promotive effects are attributed to its-induced upregulation of antioxidant enzymes-encoding genes such as *CAT*_*1*_ and *APX* [112]. Also, the HA-induced accumulation of both total flavonoids and phenols is attributed to its upregulation of the expression of phenylalanine ammonia-lyase, the critical enzyme that catalyzes the entry point of phenylpropanoid pathway through which phenolic and flavonoids are biosynthesized [113, 114]. The above HA-induced biochemical and physiological mechanisms enhance photosynthesis machinery and biomass accumulation in maize and sorghum plants, improving their growth and drought resistance. Such enhancements improve dry matter accumulation and allocation, assimilate translocation, and eventually improve maize and sorghum performance in drought-affected lands.

## Conclusion

The current investigation reports the effects of humic acid (HA) on maize and sorghum plants under drought stress and reveals differential responses across various morphological, physiological, and biochemical parameters in the two species. In the absence of HA, drought stress resulted in significant retardation in growth traits and biomass accumulation with maize generally more susceptible than sorghum. These inhibitory effects were associated with substantial disruption in photosynthetic efficiency, osmoregulation, and oxidative stress responses in both plant species. HA priming improved the overall growth and dry matter accumulation in maize and sorghum plants via maintaining higher water status and enhancing photosynthetic efficiency in the well-watered as well as droughted plants even under severe drought stress (D_2_), with significantly higher responses in maize than sorghum. Additionally, HA ameliorated the drought-induced oxidative stress by upregulating osmoprotectants and antioxidants, thus improving plant general metabolism under drought. Overall, our results highlight the potential of HA as a promising biostimulant in mitigating the adverse effects of drought stress on maize and sorghum plants, offering insights into its mechanisms of action and its application in sustainable agriculture practices and management aimed at enhancing crop resilience to drought affected lands. Future simultaneous studies at the genomic and transcriptional levels are critical to better understanding the genetic basis of the physiological differences reported in the current study.

### Electronic supplementary material

Below is the link to the electronic supplementary material.

Supplementary Material 1

## Data Availability

All data generated or analysed during this study are included in this published article and any further inquiries can be directed to the corresponding author.
