# Supplementary material for: Mitigation of drought stress in maize and sorghum by humic acid: differential growth and physiological responses
Source: BMC Plant Biol. 2024 Jun 7;24:514. doi: 10.1186/s12870-024-05184-4 (PMC11157776; doi:10.1186/s12870-024-05184-4)
Supplement: Supplementary file 1 — Supplementary Material 1 [file 12870_2024_5184_MOESM1_ESM.docx]

**Supplementary Table 1** Influence of different HA concentrations on germination and seedling growth of maize and sorghum

| Treatments | GRI | Radicle length (cm) | Plumule length (cm) | Seedling FWT  (mg) | Seedling DWT  (mg) |
| --- | --- | --- | --- | --- | --- |
| Maize | | | | | |
| Control | 39.51 ±1.66c  1.43 | 11.10 ±1.07c | 5.36 ±0.41c | 439.29 ±29.74d | 41.14 ±3.93d |
| 25 mg/l HA | 39.36 ±1.43c | 12.86 ±1.11b | 7.01 ±0.78b | 487.43 ±25.90c | 50.00 ±3.11c |
| 50 mg/l HA | 42.27 ±0.29ab | 13.50 ±0.59b | 7.24 ±0.30b | 533.29 ±17.57b | 54.29 ±3.20b |
| 100 mg/l HA | 43.62 ±0.13a | 14.96 ±1.44a | 8.53 ±0.55a | 594.86 ±26.03a | 59.43 ±3.05a |
| 150 mg/l HA | 40.62 ±1.22bc | 14.01 ±1.28ab | 8.04 ±0.95a | 564.86 ±38.44a | 55.71 ±2.14b |
| Sorghum | | | | | |
| Control | 35.51 ±1.22C | 10.06 ±0.96D | 8.54 ±0.76C | 100.43 ±5.03C | 9.86 ±0.69D |
| 25 mg/l HA | 36.17 ±2.69BC | 11.39 ±1.06C | 10.44 ±0.51B | 126.86 ±11.13B | 11.00 ±1.00C |
| 50 mg/l HA | 37.18 ±0.18BC | 12.54 ±1.00AB | 10.81 ±0.69B | 135.00 ±10.13B | 12.43 ±0.98B |
| 100 mg/l HA | 41.63 ±1.02A | 13.29 ±0.90A | 12.10 ±0.34A | 145.86 ±4.18A | 13.71 ±0.76A |
| 150 mg/l HA | 38.57 ±1.48B | 11.90 ±0.94BC | 11.00 ±0.41B | 132.29 ±8.83B | 12.48 ±0.43B |

Means ± SD (*n* = 3 for GRI and 7 for seedling indices) followed by different letters indicate significant responses whereas those followed by the same letters depict non-significant responses for the respective parameters at Fisher's test (*P* *≤* 0.05) (uppercase letters for sorghum and lowercase letters for maize). HA; humic acid, GRI; germination rate index, FWT; fresh weight, DWT; dry weight
